# Prof. Hamilton

**Published:** 1851-06

**Authors:** 


*Prof. Hamilton.*—The following letter addressed to the Editor, we publish by request of Prof. Hamilton, for the purpose of informing our correspondent and others, that Dr. *Hamilton, of Rochester*, who has charge of a hydropathic establishment, and *Dr. Frank H. Hamilton, late of Rochester and now of Buffalo*, our colleague also in the Buffalo Medical College, are two very distinct persons.

If the character of Drs. Hamilton and Halsted of Rochester, and of their respective establishments are required, we think it may be easily inferred from their advertisements, or it may be ascertained by inquiry of any respectable physician, or other citizen ot Rochester.

*Brasher Falls,*
*St. Lawrence Co., May* 15, 1851.

A. Flint, M. D.:

*Sir,*—From your connection with the Medical Journal, and the College in Buffalo, I have been induced to address you, for the purpose of inquiring in regard to the character of two rival *institutions* in Rochester for the reception of patients, especially those affected with chronic uterine complaints. The names of those having them in charge are, I think, Halsted, of one, and Hamilton, of the other.

It is currently reported in this vicinity that one is Frank H. Hamilton, M. D., formerly Prof. in Castleton and now in Buffalo; and such a *report,* even, might be the means of securing the aid of some young Physicians in this and Franklin counties for one of them, as the majority of the younger class hail Castleton as their "alma mater."

I wish to know if there be any truth in this, or if there be any such institution; or if it is only another hot-bed of Quackery?

Will you favor me with what information you can on the subject?

Respectfully yours, ———.

